# Lucidin from *Rubia cordifolia* Outperforms FDA-Approved Lapatinib as a Potential Multitargeted Candidate for Breast Cancer Signalling Proteins

**DOI:** 10.3390/ph18010068

**Published:** 2025-01-09

**Authors:** Akram Ahmed Aloqbi, Hadil Alahdal, Amany I. Alqosaibi, Mashael M. Alnamshan, Ibtesam S. Al-Dhuayan, Ahood A. Al-Eidan, Hind A. S. Alzahrani, Nouf K. ALaqeel, Fatmah Hazza Alsharif, Abeer Al Tuwaijri

**Affiliations:** 1 Department of Biological Science, Faculty of Science, University of Jeddah, Jeddah 21589, Saudi Arabia; aaaloqbi@uj.edu.sa; 2 Department of Biology, College of Science, Princess Nourah bint Abdulrahman University, Riyadh 11671, Saudi Arabia; hmalahdal@pnu.edu.sa; 3 Department of Biology, College of Science, Imam Abdulrahman bin Faisal University, P.O. Box 1982, Dammam 31441, Saudi Arabia; amgosaibi@iau.edu.sa (A.I.A.); malnamshan@iau.edu.sa (M.M.A.); ialdhuayan@iau.edu.sa (I.S.A.-D.); aeidan@iau.edu.sa (A.A.A.-E.); hizahrani@iau.edu.sa (H.A.S.A.); nalaqeel@iau.edu.sa (N.K.A.); 4 Faculty of Nursing, King Abdulaziz University, Jeddah 21461, Saudi Arabia; falsharif@kau.edu.sa; 5 Medical Genomics Research Department, King Abdullah International Medical Research Center (KAIMRC), Ministry of National Guard Health Affairs (MNGH), Riyadh 11481, Saudi Arabia; 6 Department Clinical Laboratory Sciences, College of Applied Medical Sciences, King Saud bin Abdulaziz University for Health Sciences Riyadh, Riyadh 11433, Saudi Arabia

**Keywords:** breast cancer, multitargeted therapy, Lucidin, WaterMap computations, molecular dynamics simulations

## Abstract

**Background:** Breast cancer remains a significant global health concern, with approximately 2.3 million diagnosed cases and 670,000 deaths annually. Current targeted therapies face challenges such as resistance and adverse side effects. This study aimed to explore natural compounds as potential multitargeted breast cancer therapeutics, focusing on Lucidin, an anthraquinone derived from *Rubia cordifolia*, and comparing its efficacy with Lapatinib, an FDA-approved drug. **Methods:** We performed multitargeted molecular docking studies on key breast cancer proteins using a natural compound library from ZINC. Comparative analyses of Lucidin and Lapatinib included molecular interaction fingerprints, pharmacokinetics, WaterMap computations (5 ns) to assess water thermodynamics and binding interactions, and Molecular Dynamics Simulations (100 ns) in water to evaluate complex stability and dynamic behaviour. **Results:** Lucidin demonstrated significant binding affinity and interaction potential with multiple breast cancer targets, outperforming Lapatinib in stability and binding interactions. WaterMap analysis revealed favourable hydration site energetics for Lucidin, enhancing its efficacy. The multitargeted profile of Lucidin suggests a broader therapeutic approach with potential to overcome resistance and side effects associated with Lapatinib. **Conclusions:** Lucidin shows promise as a novel, multitargeted anti-breast cancer agent with improved efficacy over Lapatinib. These findings provide a foundation for further in vitro and in vivo validation to develop Lucidin as a potential therapeutic option for breast cancer treatment.

## 1. Introduction

Breast cancer continues to be a significant global health issue, with approximately 2.3 million new cases diagnosed each year, making it the most common cancer among women [1,2,3]. In 2020 alone, it accounted for 11.7% of all cancer cases worldwide. Unfortunately, the disease also contributes to a significant number of deaths, with over 685,000 deaths annually, according to WHO’s recent estimates [3,4]. The survival rates vary widely depending on early detection and access to effective treatments, but disparities in healthcare systems lead to higher mortality rates in low- and middle-income countries [5]. This burden underscores the need for innovative and affordable therapeutic strategies. Despite the availability of targeted therapies, issues such as treatment resistance and adverse side effects remain significant hurdles [6]. Breast cancer treatment has made significant strides with the advent of targeted therapies, such as hormone therapies and human epidermal growth factor receptor-2 (HER2) inhibitors, which have improved survival rates for many patients. However, drug resistance has emerged as a significant obstacle to achieving long-term treatment success. Resistance can occur through various mechanisms, including genetic mutations, drug target alterations, and tumour microenvironment changes, which allow cancer cells to evade treatment and continue growing [7,8]. This resistance diminishes the efficacy of existing therapies and limits the options available to patients, particularly in the advanced stages of the disease [9,10]. As a result, there is growing interest in developing multitargeted drug candidates that can simultaneously attack multiple pathways involved in cancer progression to overcome the drug resistance problem [11,12,13,14].

The proteins associated with the Protein Data Bank IDs (PDBIDs) 1A52, 1N8Z, 3PXY, and 1E7U play critical roles in breast cancer progression and represent key targets in developing multitargeted therapies [15,16,17,18]. PDBID 1A52 corresponds to estrogen receptor alpha (ERα), which is a well-established driver of breast cancer, particularly in hormone receptor-positive (HR+) breast cancer. Estrogen signalling promotes the proliferation of breast cancer cells, and therapies like tamoxifen and aromatase inhibitors aim to block this pathway [15]. PDBID 1N8Z represents the structure of human epidermal growth factor receptor-2 (HER2), a receptor tyrosine kinase that is overexpressed in HER2-positive breast cancers, involved in signalling pathways that regulate cell growth and survival, and its overactivation leads to aggressive tumour behaviour [16]. Targeted therapies like trastuzumab have significantly improved outcomes for HER2-positive breast cancer patients, but resistance remains a critical issue, often due to compensatory signalling through alternative pathways. PDBID 3PXY is associated with Cyclin-dependent kinase 2 (CDK2), a protein that plays a crucial role in cell cycle regulation, particularly in controlling the transition from the G1 to the S phase. In many cancers, including breast cancer, CDK2 becomes dysregulated, allowing uncontrolled cell proliferation. Inhibiting CDK2 has been explored as a strategy to halt the growth of cancer cells [17]. PDBID 1E7U refers to PI3K alpha (Phosphoinositide 3-kinase alpha), a component of the PI3K/AKT/mTOR pathway frequently activated in breast cancer. This pathway promotes cell survival, growth, and metabolism, and its dysregulation is a common driver of tumorigenesis [18]. Mutations in PI3K are often found in hormone receptor-positive and HER2-positive breast cancers, making it a critical target for therapy. These proteins are intricately linked through the signalling networks that drive breast cancer progression. For example, both HER2 and PI3K are part of the broader network that regulates cell proliferation and survival, while ERα signalling can crosstalk with the PI3K pathway, contributing to treatment resistance. CDK2 is also regulated by upstream signals from ER and HER2 pathways, further integrating these proteins into a shared network of cancer-driving mechanisms. Designing a multitargeted drug that simultaneously inhibits these proteins could fundamentally change the landscape of breast cancer therapy. Instead of targeting a single pathway, which often leads to resistance as cancer cells adapt and activate alternative routes, a multitargeted approach would simultaneously shut down multiple survival mechanisms. For instance, targeting both HER2 and PI3K might prevent the cancer cells from bypassing HER2 inhibition by activating the PI3K pathway. Similarly, combining ERα and CDK2 inhibition could block hormone-driven cancer growth while preventing cell cycle progression. This multitargeted drug-design strategy could reduce the likelihood of resistance, as the cancer cells would have fewer options for survival [1,19,20].

Despite significant advancements in treatment, challenges such as drug resistance, severe side effects, and limited efficacy of current therapies underscore the urgent need for novel therapeutic strategies [1]. Natural compounds have emerged as promising candidates in this context due to their structural diversity and potential multitargeted therapeutic properties. Lucidin, an anthraquinone derived from *Rubia cordifolia*, has garnered attention for its diverse pharmacological activities, including anti-inflammatory, antioxidant, and anticancer properties [21]. *Rubia cordifolia* (commonly known as Indian Madder) has a long history of use in traditional medicine, where it is revered for its potential to treat various ailments, including skin disorders and cancer. Preliminary studies suggest that Lucidin exhibits potent cytotoxic activity against cancer cell lines by modulating key signalling pathways involved in tumour progression [22,23]. However, its potential as a multitargeted agent for breast cancer treatment has not been thoroughly explored, particularly in comparison to FDA-approved therapies such as Lapatinib.

In this study, we collected and prepared four crucial proteins involved in breast cancer signalling and performed multitargeted docking with the ZINC natural compound library to identify a multitarget compound and compared it to the FDA-approved drug Lapatinib. We analysed the molecular interaction fingerprint and pharmacokinetics followed by the WaterMap computation for 5 nanoseconds and MD Simulation for 100 nanoseconds to validate the preliminary computational studies.

## 2. Results and Discussion

### 2.1. Protein Preparation and Structure Assessment

**Figure 1:**
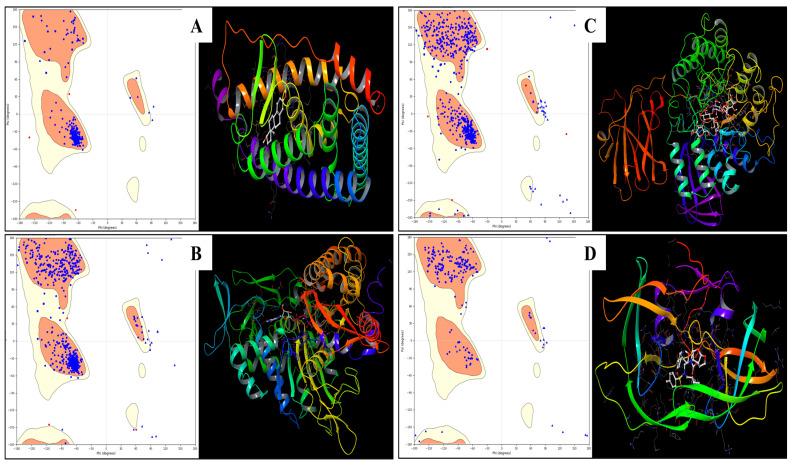
Showing the Ramachandran Plot and Prepared structures with native bound ligands for PDBIDs (**A**) 1A52, (**B**) 1N8Z, (**C**) 3PXY, and **(D**) 1E7U.

The protein structures were prepared and optimised for the bond orders, and they all were validated with the Ramachandran Plot; the binding pockets were correctly analysed. The computational analysis of four distinct proteins with PDB IDs—4AV5, 1A52, 3PXY, and 1E7U—provides insights into their structural and physicochemical properties that are relevant to protein stability, folding, and aggregation. Each protein’s AGGRESCAN results, hydrophobicity metrics, and other structural properties reveal the complex interplay between amino acid composition, surface area exposure, and molecular interactions. PDB ID 4AV5 exhibits moderate aggregation tendencies, with eight AGGRESCAN hotspots and an a3v value of 0.123. Its hydrophobic surface area (1520.10 Å^2^) and atomic contact energy (−376.24 kcal/mol) suggest a relatively compact structure with a hydrophobic moment of 0.547. The protein’s dipole moment is high (196.72 Debye), indicating significant polarity. A lower exposed hydrophobic patch energy of 605.23 and high Zeta potential (−49.29 mV) further highlight its solubility and low aggregation propensity. The secondary structure prediction indicates balanced alpha-helix and beta-sheet content, and the hydrodynamic radius (5.64 nm) aligns with a medium-sized, globular protein. PDB ID 1A52, with 10 AGGRESCAN hotspots and an a3v value of 0.157, shows a higher potential for aggregation than 4AV5. The protein’s large hydrophobic surface area (545.98 Å^2^) coupled with a more negative atomic contact energy (−321.97 kcal/mol) indicates a slightly more flexible structure. A notable negative patch energy (−60.35 kcal/mol) contributes to its stability, yet its net charge and apparent charge (−7 and 0 eV, respectively) suggest significant surface charge, influencing electrostatic interactions. The protein has a balanced secondary structure with alpha-helix and beta-sheet propensities, and its larger hydrodynamic radius (5.95 nm) hints at a more elongated conformation. PDB ID 3PXY stands out due to its high aggregation propensity, reflected by 17 AGGRESCAN hotspots, but a negative a3v value (−0.028), indicating regions resistant to aggregation. It has the largest hydrophobic surface area (2103.54 Å^2^) among the proteins and significant hydrophobic patch energies, making it prone to interaction with other hydrophobic surfaces. Despite this, it has the highest dipole moment (1993.04 Debye), which may enhance solubility. The molecular weight and high flexibility indices align with an extended, less compact structure, as confirmed by the hydrodynamic radius (55.29 nm). This protein’s high Zeta potential (−228.14 mV) suggests strong electrostatic repulsion, likely mitigating aggregation under physiological conditions. PDB ID 1E7U features 15 AGGRESCAN hotspots and an a3v value of 0.130, positioning it between 4AV5 and 1A52 regarding aggregation potential. Its moderate hydrophobic surface area (377.62 Å^2^) and relatively high atomic contact energy (−465.16 kcal/mol) indicate a stable, yet somewhat flexible, protein. The hydrophobic moment (0.583) and hydrophobic patch energy (1125.82 kcal/mol) suggest well-distributed hydrophobic regions, contributing to its intermediate solubility. The secondary structure elements predict balanced alpha-helix and beta-sheet content, and a Zeta potential of −48.82 mV suggests moderate electrostatic stability, reducing the risk of aggregation. The four proteins exhibit diverse physicochemical properties affecting their stability, solubility, and aggregation behaviour. Both 4AV5 and 1E7U show moderate aggregation tendencies and structural stability, while 1A52 presents a higher aggregation potential but increased electrostatic interactions. In contrast, 3PXY displays the highest aggregation potential and significant hydrophobic interaction and electrostatic stabilisation mechanisms. These findings provide valuable insight into the structure-function relationship of these proteins, offering potential strategies for modulation of their stability in biological or industrial applications. Further, the detailed energy produced in each case is shown in Table 1 to provide a better understanding, and Figure 1 provides a clear understanding of the Ramachandran plot and the structures of the proteins.

### 2.2. Multitargeted Molecular Docking and MM/GBSA Studies

**Figure 2:**
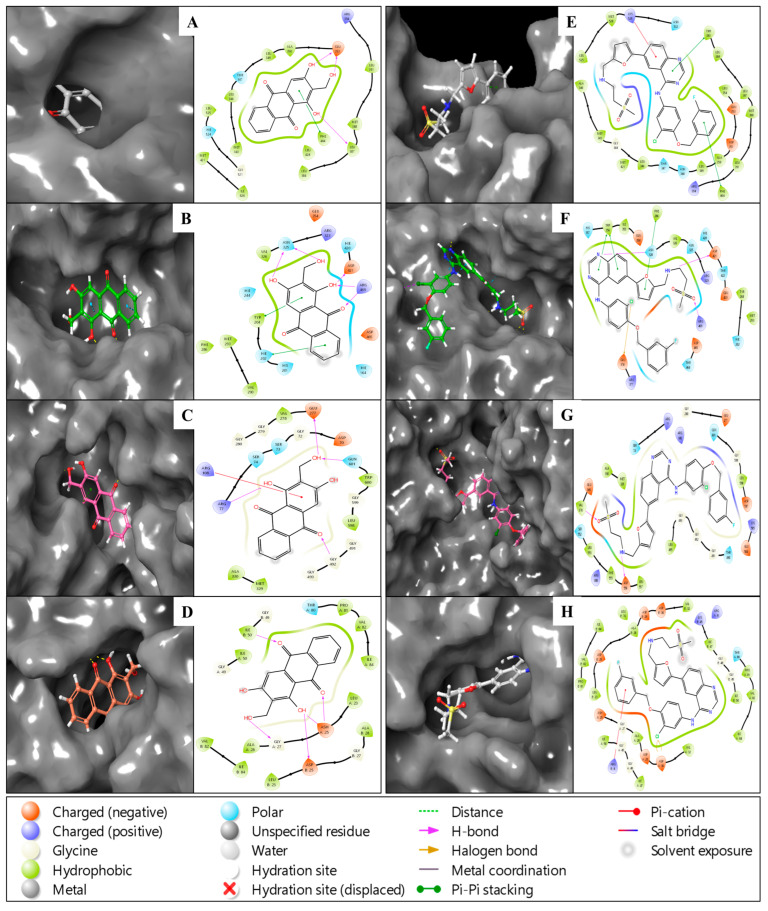
Showing the docked poses in 3D and 2D for PDBIDs—(**A**) 1A52, (**B**) 1N8Z, (**C**) 3PXY, and (**D**) 1E7U in complex with Lucidin and (**E**) 1A52, (**F**) 1N8Z, (**G**) 3PXY, and (**H**) 1E7U in complex with control drug Lapatinib.

The multitargeted docking strategy identified Lucidin, binding with the best scores for all four complexes, and so this was chosen to prove its multitargeted potency and was appropriately analysed. The multitargeted docking strategy identified Lucidin as a potent ligand capable of binding to multiple protein targets associated with breast cancer subtypes, including HER2-positive (HER2+), estrogen receptor-positive (ER+), and triple-negative breast cancer (TNBC). The analysis revealed Lucidin’s binding potential by calculating docking scores, MM/GBSA binding free energies, and additional energy components such as van der Waals (vdW) interactions, electrostatic contributions, and hydrogen-bond interactions. Lucidin demonstrated strong binding to the estrogen receptor (PDBID: 1A52) with a docking score of −9.90 kcal/mol and an MM/GBSA binding energy of −46.51 kcal/mol. Significant vdW contributions of −1066.30 kcal/mol further supported the complex’s stability. Hydrogen bond interactions with residues of GLU353 and LEU387 involved three hydroxyl groups (OH) of Lucidin, while a π-π stacking interaction with PHE404 stabilised the complex via the benzene ring of the ligand. These interactions suggest that Lucidin may be an effective modulator for ER+ breast cancer (Figure 2). Lucidin showed binding potential to HER2 (PDBID: 1N8Z), with a docking score of −6.59 kcal/mol and an MM/GBSA binding energy of −25.12 kcal/mol. Key interactions included five hydrogen bonds with residues of ASN325, ASP421, and ARG469 and π-π stacking with HIE202 and TYR204. Electrostatic interactions also played a role, contributing −3236.45 kcal/mol to the total binding energy. These results indicate Lucidin’s potential utility for HER2+ breast cancer therapies. Lucidin exhibited moderate binding to CDK2 (PDBID: 3PXY) with a docking score of −5.65 kcal/mol and an MM/GBSA binding energy of −31.82 kcal/mol. Hydrogen bond formation with ARG77, GLU277, GLN601, and GLY492 and a π-cation interaction involving ARG108 were observed. The vdW contribution of −2752.87 kcal/mol reinforced the ligand’s binding affinity. This interaction profile supports Lucidin’s versatility as a multitargeting agent. Lucidin achieved a docking score of −7.62 kcal/mol and an MM/GBSA binding energy of −44.92 kcal/mol with PI3K (PDBID: 1E7U). Multiple hydrogen bonds were observed with ASP25, ASH25, and GLY27, as well as π-cation interactions with ASH25 and ILE50. The vdW energy of −958.29 kcal/mol contributed significantly to the binding. This interaction suggests Lucidin’s potential effectiveness against TNBC, where PI3K inhibitors are often targeted (Figure 2).

To benchmark these findings, Lapatinib, an FDA-approved breast cancer drug, was docked with the same targets. Lapatinib with Estrogen Receptor (PDBID: 1A52) produced a docking score of −7.31 kcal/mol and an MM/GBSA binding energy of −38.51 kcal/mol. π-π stacking interactions with TRP383 and PHE404, along with a π-cation contact with LYS529, were observed. These values, while significant, are weaker when compared to Lucidin’s performance with the same target. Lapatinib with HER2 (PDBID: 1N8Z) docking score was −3.57 kcal/mol, with an MM/GBSA energy of −28.39 kcal/mol. The interaction involved three hydrogen bonds with ASN328, ASP421, and ARG469, as well as three π-π stacking contacts with PHE286 and TRP356. While comparable to Lucidin, Lapatinib’s lower scores indicate a relatively weaker binding affinity. Lapatinib with CDK2 (PDBID: 3PXY) scored −3.61 kcal/mol for docking and −43.31 kcal/mol for MM/GBSA binding energy. Key interactions included hydrogen bonding with ASP556 and VAL370 and vdW contributions of −2775.54 kcal/mol. These values suggest that while Lapatinib interacts effectively with CDK2, its overall binding affinity is lower than Lucidin’s. For PI3K (PDBID: 1E7U), Lapatinib achieved a docking score of −7.96 kcal/mol and an MM/GBSA energy of −58.84 kcal/mol, with interactions involving halogen bonds with ASP30 and a π-cation contact with ARG8 (Figure 2). Despite its high MM/GBSA energy, the overall interaction profile aligns closely with Lucidin. Lucidin consistently demonstrated strong binding across all targets, with superior docking and binding energies compared to Lapatinib in most cases. These results highlight its potential as a multitargeted agent for HER2+, ER+, and TNBC breast cancer subtypes. Table 2 summarises all calculated energies, while Figure 2 illustrates the 2D and 3D confirmations, providing a comprehensive view of Lucidin’s efficacy and versatility as a candidate for further studies.

*Rubia cordifolia* (Indian Madder) has gained attention for its diverse pharmacological properties and anticancer potential. Lucidin, an anthraquinone derivative isolated from *Rubia cordifolia*, has been identified as a compound of interest due to its reported biological activities, including anti-proliferative effects on various cancer cell lines. However, its potential as a multitargeted therapeutic agent in breast cancer has not been thoroughly investigated. Multitargeted therapies are increasingly recognised as a promising approach to overcoming drug resistance, as they can simultaneously inhibit multiple pathways involved in cancer progression. This is particularly relevant in the context of breast cancer, a complex and heterogeneous disease characterised by various molecular subtypes, each with distinct genetic and phenotypic profiles. The plasticity of cancer cells and the redundancy of signalling pathways often lead to the development of resistance, reducing the efficacy of monotherapies. Lapatinib, a well-established dual tyrosine kinase inhibitor targeting both HER2 and EGFR, has been widely used in the treatment of HER2-positive breast cancer. However, issues such as acquired resistance and cardiotoxicity have limited its long-term efficacy, highlighting the need for alternative or complementary therapies. This study aims to evaluate the potential of Lucidin as a multitargeted therapeutic candidate for breast cancer by conducting a comparative analysis with Lapatinib. We employed molecular docking, WaterMap, and molecular dynamics simulation studies to investigate the binding interactions, hydration site energetics, and dynamic behaviour of Lucidin and Lapatinib within relevant protein targets. Natural products have historically been a rich source of therapeutic agents, with many current anticancer drugs derived from natural compounds. Lucidin represents a promising candidate for breast cancer treatment due to its potential to interact with multiple molecular targets. Previous studies have suggested that Lucidin exhibits various biological activities, including anti-inflammatory, antimicrobial, and anticancer effects. However, the specific mechanisms by which Lucidin might exert its anticancer properties, particularly in the context of breast cancer, remain largely unexplored. This investigation provides insights into the molecular basis of Lucidin’s activity and offers a comparative perspective with an established drug, Lapatinib, potentially paving the way for developing more effective and safer breast cancer therapies. Through these computational approaches, we seek to elucidate the mechanisms by which Lucidin may exert its anticancer effects and assess its potential as a therapeutic agent in breast cancer treatment. The insights gained from this research could contribute to developing novel therapeutic strategies that leverage the multitargeting capabilities of natural compounds like Lucidin, ultimately improving outcomes for patients with breast cancer.

### 2.3. Molecular Interaction Fingerprints and Pharmacokinetics Assesment

**Figure 3:**
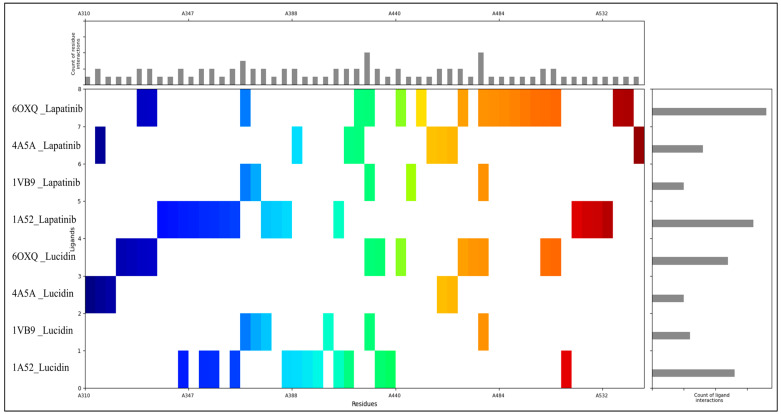
Showing the Molecular Interaction Fingerprints (MIFs) for PDBIDs—1A52, 1N8Z, 3PXY, and 1E7U in complex with Lucidin and with control drug Lapatinib.

The MIFs of proteins interacting with Lucidin (our identified candidate) and Lapatinib (FDA-approved drug) reveal the role of various amino acid residues in stabilising these protein-ligand complexes through various interaction types. Hydrophobic residues, such as leucine (19 occurrences), methionine (7 occurrences), isoleucine (6 occurrences), valine (6 occurrences), alanine (4 occurrences), proline (2 occurrences), phenylalanine (2 occurrences), and tryptophan (2 occurrences), play a critical role in forming van der Waals and hydrophobic interactions. These residues create a stable, non-polar environment within the binding pocket, which helps to stabilise the ligand by burying it away from the solvent. For instance, leucine, methionine, and isoleucine engage in van der Waals interactions, while aromatic residues like phenylalanine and tryptophan may also participate in π-π stacking or π-cation interactions with the ligands, contributing to the overall stabilisation of the complex. Polar uncharged residues, including glutamine (two occurrences), serine (two occurrences), and threonine (two occurrences), contribute primarily through hydrogen bonding. These residues help position the ligand within the binding site by interacting with hydrogen bond donors or acceptors on the ligand. For example, the amide side chain of glutamine and the hydroxyl groups of serine and threonine facilitate specific polar contacts, which are crucial for ligand orientation and binding specificity. Positively charged residues, such as lysine (six occurrences) and arginine (one occurrence), are involved in electrostatic interactions, often forming salt bridges with negatively charged groups on the ligands. Similarly, arginine’s guanidinium group forms strong electrostatic interactions and can engage in multiple interactions simultaneously. Negatively charged residues, including glutamic acid (10 occurrences) and aspartic acid (6 occurrences), participate in electrostatic interactions and hydrogen bonding. Histidine (one occurrence) and glycine (four occurrences) are also important in the interaction profiles. Histidine, with its imidazole ring, is versatile in forming hydrogen bonds and electrostatic interactions, depending on the pH. It can act as a hydrogen bond donor or acceptor and may also participate in stacking interactions with aromatic groups in the ligand. Glycine, although not directly involved in hydrophobic or electrostatic interactions due to its lack of a side chain, provides flexibility to the protein structure, allowing other residues to orient themselves for optimal ligand binding. The combination of hydrophobic, polar, and charged residues provides a balanced network of interactions that stabilise Lucidin and Lapatinib in the binding pockets of the target proteins. Hydrophobic interactions significantly stabilise the ligand within non-polar pockets, while polar and charged residues ensure proper orientation and specificity through hydrogen bonding and electrostatic interactions. This rich landscape of molecular interactions is key to the effectiveness of Lucidin and Lapatinib as drug candidates. Figure 3 shows a better representation and distribution of residues forming interactions and its counts and the number of interactions formed by the ligands.

The pharmacokinetic evaluation of Lucidin, our identified multitargeted drug candidate, reveals several promising characteristics that suggest it may serve as an effective treatment option for breast cancer when compared to the FDA-approved drug Lapatinib. The QikProp predictions demonstrate that Lucidin possesses favourable properties across various parameters, indicating its potential for therapeutic use. Lucidin and Lapatinib meet Lipinski’s Rule of Five, which is essential for predicting oral bioavailability, though Lucidin has a distinct advantage with zero violations, compared to two violations for Lapatinib, suggesting that Lucidin is likely to exhibit superior oral bioavailability. Furthermore, the human oral absorption of Lucidin is 65.73%, falling within an acceptable range, though slightly lower than Lapatinib’s absorption at 80.087%. Despite this, Lucidin remains in a favourable position for oral drug delivery. In terms of solubility and lipophilicity, Lucidin demonstrates significantly better aqueous solubility, with a QPlogS value of −3.159, as opposed to Lapatinib’s value of −8.299. This superior solubility is crucial for enhancing absorption and systemic distribution. Lucidin’s octanol-water partition coefficient (QPlogPo/w) of 0.56 indicates a better balance between hydrophilicity and hydrophobicity, favouring drug distribution and uptake. In contrast, Lapatinib’s QPlogPo/w value of 5.914 suggests it is more lipophilic, which could limit its bioavailability in aqueous environments. Lucidin also presents a less extensive metabolic profile, with three predicted metabolic sites compared to Lapatinib’s six. This could potentially lead to better metabolic stability and a longer half-life, reducing the risk of forming potentially toxic metabolites. The fewer metabolic sites further enhance Lucidin’s potential for consistent therapeutic activity in vivo. Regarding cardiotoxicity risks, Lucidin presents a lower risk of hERG channel inhibition compared to Lapatinib, which is crucial for minimising adverse cardiac effects. Lucidin’s QPlogHERG value of −4.468, though near the threshold of concern, is considerably safer than Lapatinib’s value of −8.159, which indicates a higher risk of cardiac side effects. The molecular weight of Lucidin (270.241 Da) is significantly lower than that of Lapatinib (581.06 Da), which can aid in better tissue penetration and pharmacokinetic behaviour. Lucidin’s polar surface area (PSA) of 115.139 Å^2^, compared to Lapatinib’s 98.987 Å^2^, strikes an optimal balance between solubility and membrane permeability, contributing to its potential as an effective therapeutic agent. Lucidin’s CNS inactivity, as reflected by a CNS score of −2, indicates limited central nervous system penetration, reducing the risk of neurotoxic side effects. On the other hand, Lapatinib’s CNS score of 1 implies a higher likelihood of CNS-related adverse effects, making Lucidin a safer alternative for chronic use. In terms of molecular flexibility, Lucidin has fewer rotatable bonds (4) than Lapatinib (10), which correlates with a more rigid molecular structure. This rigidity can enhance receptor binding specificity, potentially improving target affinity and selectivity—key aspects of multitargeted cancer therapies. Furthermore, Lucidin’s low blood-brain barrier permeability (QPlogBB = −1.494) minimises the risk of off-target effects in the central nervous system, an advantage over Lapatinib. Lucidin also exhibits a lower dipole moment (2.712 Debye), indicating its reduced polarity compared to Lapatinib (9.289 Debye), favouring selective interaction with cancer cell membranes while sparing healthy tissues. The pharmacokinetics results show that our identified drug candidate, Lucidin, has promising pharmacokinetic properties, including favourable solubility, metabolic stability, reduced cardiotoxicity, and minimal central nervous system penetration. These attributes suggest that Lucidin has the potential to serve as a highly effective multitargeted therapeutic agent for breast cancer, with a safety and efficacy profile that surpasses that of Lapatinib. Table 3 provides a detailed view of descriptors, QikProp standard values, and computed pharmacokinetics of Lucidin and Lapatinib.

### 2.4. WaterMap Analysis

The WaterMap results offer insightful details on the hydration thermodynamics at the protein-ligand interface, highlighting the differences in water displacement upon ligand binding. The data show varying degrees of hydration energies (ΔG values), and these differences can provide valuable information about ligand efficacy and binding preferences. PDB ID 1A52 with Lucidin has shown an ΔG of hydration, revealing a total energy change of −9.014 kcal/mol in the first hydration shell (Trans1). This indicates that the displacement of water molecules is highly favourable energetically, suggesting that Lucidin interacts effectively with the protein, leading to the release of structured water in a highly exothermic manner. Trans2 and Trans3 values (1.789 and 0.07 kcal/mol, respectively) show weak energy contributions, indicating that water in the second and third hydration shells does not contribute significantly to the binding interaction, highlighting Lucidin’s strong binding in the first shell. The WaterMap results, obtained using water as the solvent environment, provide detailed insights into hydration thermodynamics at the protein-ligand interface. While it is acknowledged that cancer cells often exist in a slightly acidic microenvironment, water remains a standard and representative model for hydration studies due to its ubiquity and simplicity in experimental and computational simulations. This approach ensures consistency, reproducibility, and compatibility with established thermodynamic data. In this study, water was chosen to focus on the fundamental hydration energies (ΔG values) and their contributions to binding interactions. Adjusting to an acidic pH could alter hydration shell dynamics and energetics, but the choice of water allows for a baseline understanding of Lucidin’s binding properties. Future investigations may involve simulating acidic conditions to assess pH-specific effects, but the current findings on hydration shell contributions are robust indicators of Lucidin’s strong interaction with the protein, particularly in the first hydration shell. PDB ID 1A52 with Lapatinib shows that Lapatinib displays a much more negative ΔG for Trans1 (−12.806 kcal/mol), indicating even stronger and more favourable water displacement upon binding, possibly due to better fitting or complementary interactions with the binding site. The Trans2 (−3.661 kcal/mol) and Trans3 (−2.785 kcal/mol) values also suggest that Lapatinib displaces water more efficiently in deeper hydration shells than Lucidin, which might indicate stronger overall binding energy. PDB ID 1N8Z with Lucidin shows that the Lucidin exhibits relatively weak negative energy for Trans1 (−1.069 kcal/mol), indicating marginal water displacement in the immediate binding site. However, the Trans2 (16.33 kcal/mol) and Trans3 (9.906 kcal/mol) values are positive and significantly high, implying that water molecules are retained or more structured in these shells. This could indicate that Lucidin’s interaction at this binding site is less efficient or that water remains tightly bound, reducing its overall binding energy. PDB ID 1N8Z with Lapatinib shows that ΔG values for Lapatinib in 1N8Z show a weak Trans1 value (−1.492 kcal/mol), indicating weak initial water displacement. However, Trans2 (9.32 kcal/mol) and Trans3 (11.829 kcal/mol) show strong positive values, indicating that, like Lucidin, the hydration layers remain structured and less water is displaced, suggesting that neither Lucidin nor Lapatinib efficiently disrupts the water network in this binding site, making this a less favourable pocket for interaction.

PDB ID 3PXY with Lucidin shows that Lucidin demonstrates a weakly negative ΔG for Trans1 (−1.206 kcal/mol), indicating minimal water displacement in the immediate binding region. The subsequent hydration layers also exhibit moderate positive values (Trans2 = 3.754 kcal/mol and Trans3 = 3.069 kcal/mol), indicating that water is relatively structured in these layers. This may suggest that Lucidin does not bind as tightly or efficiently at this site as others, with water molecules playing a more structured role. PDB ID 3PXY with Lapatinib shows that the binding of Lapatinib to 3PXY shows a similar weak Trans1 ΔG value (−1.231 kcal/mol), indicating minimal water displacement in the first hydration layer, but the Trans2 (6.927 kcal/mol) and Trans3 (6.865 kcal/mol) values suggest that water molecules are more structured or retained in the deeper hydration shells. This indicates that Lapatinib also does not displace much water at this binding site, similar to Lucidin. PDB ID 1E7U with Lucidin shows that the Lucidin has a slightly positive Trans1 value (0.252 kcal/mol), indicating that water displacement is thermodynamically unfavourable in the first hydration layer. The Trans2 (−4.484 kcal/mol) and Trans3 (−0.124 kcal/mol) values are negative, suggesting that Lucidin binds better in the deeper hydration layers, where it manages to displace structured water more favourably, even though the initial interaction in the first shell is not as strong. PDB ID 1E7U with Lapatinib shows that the Lapatinib demonstrates a strong negative Trans1 ΔG value (−1.321 kcal/mol), indicating that the first hydration shell is displaced more favourably than for Lucidin. The Trans2 (−5.092 kcal/mol) and Trans3 (−1.693 kcal/mol) values further indicate that Lapatinib disrupts structured water more effectively in the deeper hydration layers. Lapatinib exhibits better water displacement and thus may form stronger interactions in this binding pocket than Lucidin.

**Figure 4:**
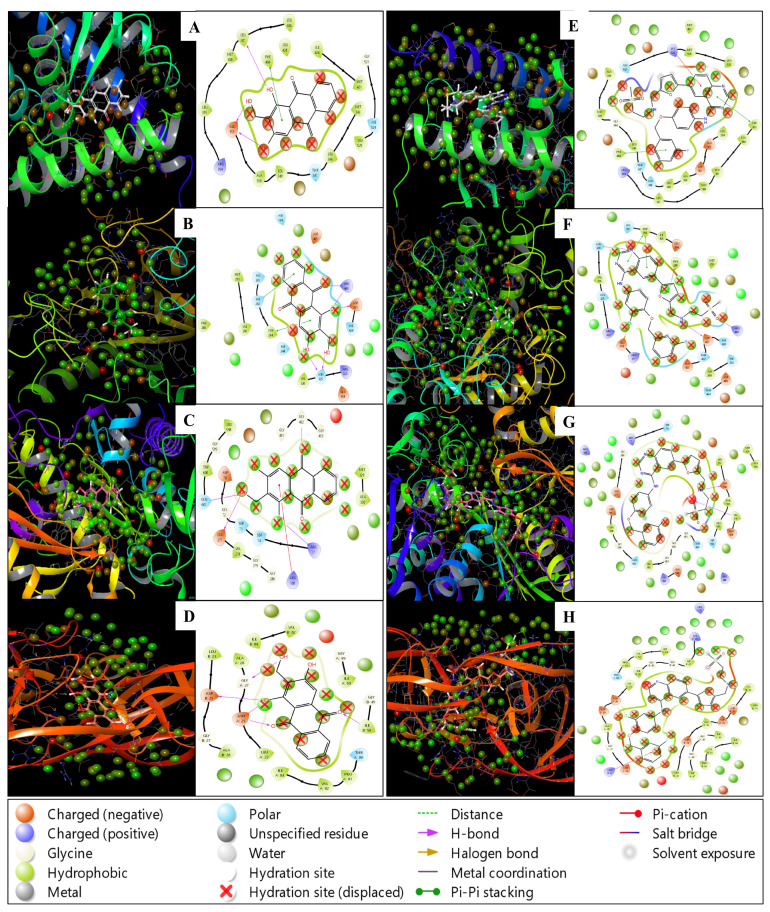
Showing the WaterMap results in 3D and 2D for PDB IDs (**A**) 1A52, (**B**) 1N8Z, (**C**) 3PXY, and (**D**) 1E7U in complex with Lucidin and (**E**) 1A52, (**F**) 1N8Z, (**G**) 3PXY, and (**H**) 1E7U in complex with control drug Lapatinib.

Water Displacement Efficiency reveals that Lapatinib generally shows more negative ΔG values across the hydration shells, especially in Trans1, indicating that it displaces water more efficiently and forms stronger interactions with protein targets than Lucidin. This suggests that Lapatinib may have a tighter binding affinity in most cases, contributing to its FDA approval status. While Lucidin may not displace water as effectively as Lapatinib, it still shows promising results, especially in the 1A52 complex, where its first shell water displacement is highly favourable. This suggests that Lucidin has potential as a multitargeted therapeutic agent, but its interaction with water may need optimisation for specific binding sites. Lucidin and Lapatinib show varying water displacement efficiencies depending on the protein target, highlighting the importance of context-specific binding characteristics. For example, while Lucidin performs well in the 1A52 binding site, it appears less effective in the 1N8Z and 3PXY complexes, where water retention remains significant. The WaterMap results highlight key differences in the hydration thermodynamics between Lucidin and Lapatinib across various protein binding sites. While Lapatinib generally shows stronger water displacement, Lucidin’s favourable interaction in specific contexts demonstrates its potential as a multitargeted drug candidate for breast cancer. Further optimisation of its water displacement profile could enhance its efficacy. Further, Figure 4 shows a comprehensive view of WaterMap to make better representations.

### 2.5. Molecular Dynamics Simulations Studies

The system builder has generated 36011, 61859, 86400, 25340, 36023, 61862, 86394, and 25361 atoms for 1A52, 1N8Z, 3PXY, and 1E7U in complex with Lucidin and Lapatinib, respectively. These files were loaded and kept for 100 nanoseconds for the production run, and the results were analysed for the deviation, fluctuation, and intermolecular interactions. The MD simulation results provide insights into the box dimensions that define the boundaries of the simulated systems for both Lucidin and Lapatinib when bound to different protein targets. These dimensions, measured in Ångströms, reflect the spatial requirements and environment for each ligand-protein interaction during the simulation. In the case of the 1A52 protein complex, Lucidin had box dimensions of 92.66 Å (x-axis), 69.64 Å (y-axis), and 55.81 Å (z-axis), creating a moderately sized system. Lapatinib, when bound to the same protein, had nearly identical dimensions of 92.64 Å (x), 69.62 Å (y), and 55.80 Å (z), indicating that both ligands require similar spatial parameters in this binding environment. For the 1N8Z complex, the simulation box for Lucidin was larger, especially along the y-axis, with dimensions of 87.09 Å, 101.55 Å, and 69.70 Å for the x, y, and z axes, respectively. Lapatinib’s box was slightly smaller, measuring 86.99 Å, 101.43 Å, and 69.62 Å, suggesting that both ligands occupy similar volumes in this system, though Lucidin might interact with a more extended region of the protein or have slightly different conformations during binding. In the 3PXY protein complex, Lucidin had box dimensions of 82.40 Å, 83.69 Å, and 125.55 Å, with a larger z-axis, possibly reflecting an elongated interaction region. Lapatinib’s box dimensions were very similar, at 82.46 Å, 83.76 Å, and 125.65 Å, indicating that both ligands fit similarly within this binding environment. For the 1E7U protein complex, Lucidin’s box dimensions were 75.13 Å, 60.19 Å, and 56.09 Å, representing a more compact system. Lapatinib had slightly smaller dimensions of 75.02 Å, 60.10 Å, and 56.01 Å, showing that both ligands require nearly identical space in this complex. The MD simulations demonstrate that Lucidin and Lapatinib occupy similar volumes in their respective protein-ligand complexes across all targets, with only minor variations in the dimensions. These results suggest that Lucidin, like Lapatinib, is well-suited to fit within various protein environments, supporting its potential as a multitarget drug candidate for therapeutic applications. Further, the detailed results for deviation, fluctuations, and intermolecular interactions and their discussion are as follows.

#### 2.5.1. Root Means Square Deviation (RMSD)

**Figure 5:**
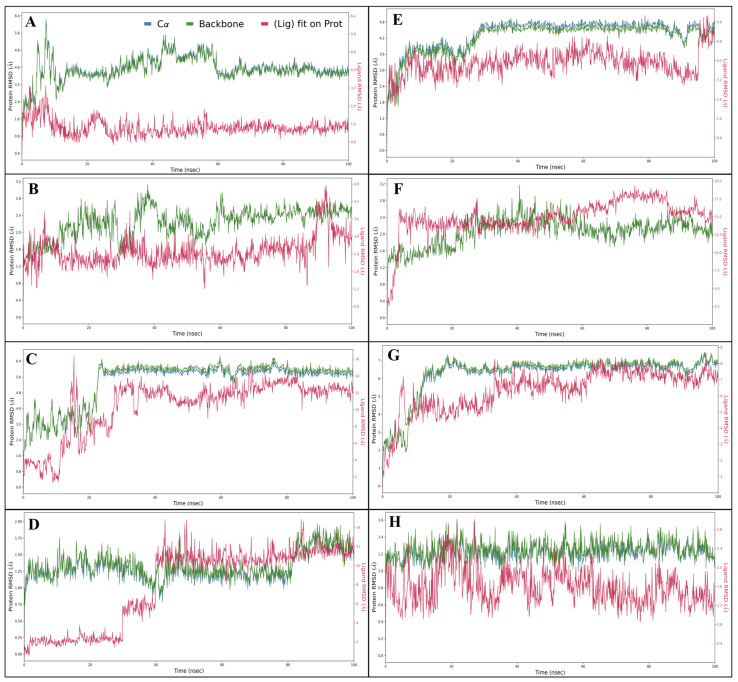
Showing the Root Mean Square Deviation (RMSD) produced during 100 nanoseconds MD Simulation for PD BIDs (**A**) 1A52, (**B**) 1N8Z, (**C**) 3PXY, and (**D**) 1E7U in complex with Lucidin and (**E**) 1A52, (**F**) 1N8Z, (**G**) 3PXY, and (**H**) 1E7U in complex with control drug Lapatinib.

Root Mean Square Deviation (RMSD) measures the average deviation between the positions of atoms in protein-ligand (P-L) complexes over time. It indicates stability; lower RMSD values suggest a more stable interaction, while higher values may indicate structural fluctuations, reflecting the binding affinity and dynamics of the complex. The complex of Estrogen Receptor (PDB ID 1A52) with Lucidin stated that the protein shows deviation at 1.86 Å while ligand deviation occurs at 1.93 Å at 0.30 ns. Throughout the simulation, steady performance was observed, with the RMSD of the complex showing acceptable variations when excluding the initial timeframe. Notably, the protein exhibited a deviation of 3.72 Å, and the ligand showed 1.34 Å at 100 nanoseconds. The complex of protein Human HER2 (PDB ID 1N8Z) with Lucidin produced a deviation of protein 1.20 Å, while in the case of ligand 1.97 Å, this occurred at 0.10 ns. The entire simulation exhibits a stable performance. At 100 nanoseconds, the protein deviated at 2.57 Å, and the ligand deviated at 2.86 Å, and when the initial period is neglected, the RMSD of the protein and ligand shows acceptable deviations. The complex of Triphosphate Diphosphohydrolase 3 (NTPDase3) (PDB ID 3PXY) with Lucidin shows protein deviation at 4.34 Å while ligand deviation is observed at 3.59 Å at 3.40 ns. Throughout the simulation, steady performance was observed, with the RMSD of the complex showing acceptable variations when excluding the initial timeframe. Notably, the protein exhibited a deviation of 5.84 Å, and while the ligand exhibited 11.96 Å deviation at 100 nanoseconds. The complex of phosphoinositide 3-kinase (PDB ID 1E7U) and Lucidin showed that the protein exhibits deviation at 0.97 Å, while ligand deviation occurs at 1.59 Å at 0.10 ns. Throughout the simulation, steady performance was observed, with the RMSD of the complex showing acceptable variations when excluding the initial timeframe. Notably, the protein exhibited a deviation of 1.56 Å and the ligand exhibited deviation of 11.41 Å at 100 nanoseconds. For the Estrogen Receptor (PDB ID 1A52) in complex with Lapatinib, the protein shows deviation at 2.94 Å while the ligand shows deviation at 3.69 Å at 0.90 ns. Throughout the simulation, steady performance was observed, with the RMSD of the complex showing acceptable variations when excluding the initial timeframe. Notably, the protein exhibited a deviation of 4.82 Å, and the ligand showed 5.09 Å at 100 nanoseconds. In the complex Human HER2 (PDB ID 1N8Z) with Lapatinib, the deviation of the protein occurred at 0.88 Å, while for the ligand, deviation occurred at 3.16 Å at 0.10 ns. The entire simulation exhibits a stable performance. At 100 nanoseconds, the protein deviated at 1.93 Å, and the ligand deviated at 14.35 Å, and when the initial period is neglected, the RMSD of the protein and ligand shows acceptable deviations. The complex of Triphosphate Diphosphohydrolase 3 (NTPDase3) (PDB ID 3PXY) with Lapatinib shows protein deviation at 4.85 Å while ligand deviation occurs at 5.09 Å at 9.50 ns. Throughout the simulation, steady performance was observed, with the RMSD of the complex showing acceptable variations when excluding the initial timeframe. Notably, the protein exhibited a deviation of 6.84 Å, and the ligand exhibited a deviation of 7.11 Å at 100 nanoseconds. The complex of phosphoinositide 3-kinase (PDB ID 1E7U) and Lapatinib revealed that protein exhibits deviation at 0.88 Å while the ligand deviation occurs at 1.44 Å at 0.10 ns. Throughout the simulation, steady performance was observed, with the RMSD of the complex showing acceptable variations when excluding the initial time frame. Notably, the protein exhibited a deviation of 1.05 Å and the ligand 1.28 Å at 100 nanoseconds. Further, Figure 5 has details of RMSD for each case. The consistent observation across different proteins demonstrates that the complexes formed with Lucidin are notably more stable than those with Lapatinib. This stability is critical for drug design, as a more stable protein-ligand complex generally indicates stronger binding affinities and potentially enhanced therapeutic efficacy. The reduced deviation in the Lucidin complexes suggests that they may maintain their conformation upon binding, leading to improved pharmacodynamics and pharmacokinetics. Furthermore, the favourable RMSD profiles observed with Lucidin imply that it may exhibit a lower likelihood of displacement or dissociation in a biological environment, thus increasing the potential for therapeutic effectiveness. This stability can also result in fewer off-target effects as the binding site geometry is maintained, thereby enhancing specificity. This characteristic makes Lucidin a promising candidate for further exploration in drug design, especially considering the implications of stability on binding affinity and therapeutic potential. Future studies should focus on elucidating the mechanisms of these interactions at a molecular level, potentially leading to the development of more effective therapeutic agents.

#### 2.5.2. Root Means Square Fluctuation (RMSF)

**Figure 6:**
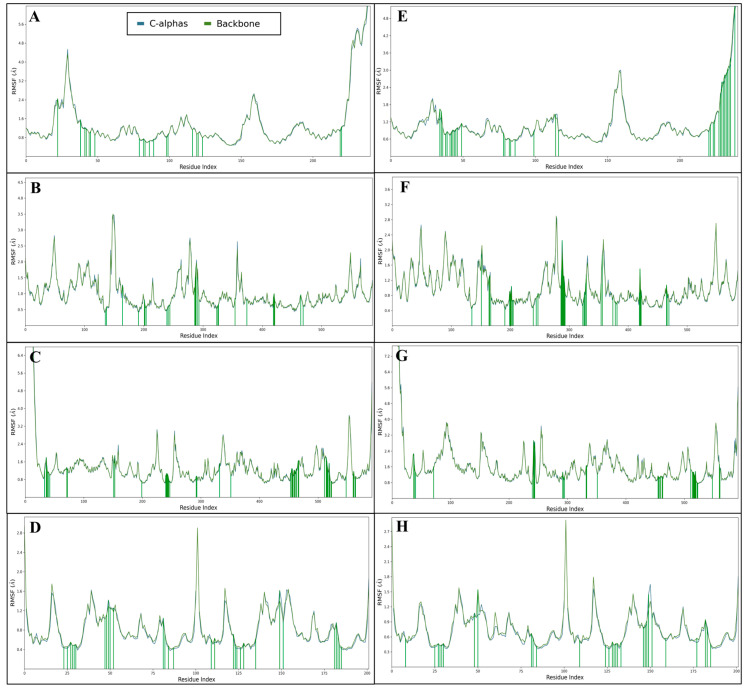
Showing the Root Mean Square Fluctuations (RMSF) produced during 100 nanoseconds MD Simulation for PDB IDs (**A**) 1A52, (**B**) 1N8Z, (**C**) 3PXY, and (**D**) 1E7U in complex with Lucidin and (**E**) 1A52, (**F**) 1N8Z, (**G**) 3PXY, and (**H**) 1E7U in complex with control drug Lapatinib.

Root Mean Square Fluctuation (RMSF) quantifies the flexibility of individual residues in a protein-ligand (P-L) complex during molecular dynamics simulations. It measures the average deviation of each atom’s position from its average position over time. Higher RMSF values indicate greater mobility and flexibility, while lower values suggest more rigid regions, helping to identify critical areas for ligand binding and protein stability. PDB ID 1A52 in complex with Lucidin has shown a few residues fluctuating; the most fluctuating residues are PRO333-PRO336 and LYS531-LEU544. At the same time, the residues that have formed interactions to make the complex stable are LEU327, MET343, LEU346, THR347, LEU349, ALA350, GLU353, LEU384, LEU387, MET388, LEU391, ARG394, LEU403, PHE404, MET421, ILE424, PHE425, LEU428, HIS524, and LEU525. PDB ID 1N8Z in complex with Lucidin has shown a few residues fluctuating; the most fluctuating residue is SER147-PRO151. At the same time, many residues have formed interactions to make the complex stable, including GLN135, PHE137, HIS164, TYR191, HIS201, HIS202, TYR204, ASP239, VAL241, HIS244, PHE286, ALA287, VAL288, VAL290, MET293, ARG323, ASN325, VAL326, GLU354, TYR374, SER419, HIS420, ASP421, ASP465, and ARG469. PDB ID 3PXY in complex with Lucidin has shown none of the residues fluctuating beyond 2 Å and shows one of the most stable complex by forming interactions with several residues that make the complex stable, including LEU23, ASP25, GLY27, ALA28-ASP30, ILE47-ILE50, GLY52, PRO81, VAL82, ILE84, ARG87, ARG8, LEU10, GLU21, ALA22, LEU23, ASP25, GLY27, GLU34, GLY48, ILE50, THR80, PRO81, VAL82, ASN83, and ILE84. PDB ID 1E7U in complex with Lucidin has shown a few fluctuating residues, including THR38-LYS46. At the same time, many residues have formed interactions to make the complex stable, including ASP70, SER73, SER74, SER75, ARG77, ASN79, ARG108, PHE109, THR188, GLY190, GLU236, GLU277, VAL278, GLY279, GLY280, ALA281, SER282, GLN284, MET329, ALA330, GLU369, ARG388, THR490, GLY492, GLY493, MET494, ALA496, ALA497, ASN499, THR500, ASP503, HIS504, GLN548, ASN551, SER552, LEU553, ASN554, ASP556, LEU557, LYS559, THR560, LYS585, ASP597, LEU598, TRP600, and GLN601. PDB ID 1A52 in complex with Lapatinib has shown a few fluctuating residues, including SER464, LEU539, and LEU540-LEU544. At the same time, several residues have formed interactions to make the complex stable, including GLU339, ALA340, SER341, MET343, GLY344, LEU346, THR347, ASN348, LEU349, ALA350, ASP351, LEU354, TRP383, LEU384, LEU387, MET388, LEU391, PHE404, GLU419, MET421, LEU525, TYR526, MET528, LYS529, ASN532, VAL533, VAL534, PRO535, LEU536, TYR537, ASP538, LEU539, LEU540, and MET543. PDB ID 1N8Z in complex with Lapatinib has shown a few fluctuating residues, and the most fluctuating residues include SER47-GLU50, PRO89-GLU92, LYS277-SER279, VAL288, ASP358, and GLU547-GLY550. At the same time, many residues have formed interactions to make the complex stable, including GLN135, PRO151, HIS164, ASP165, PHE167, TYR191, PRO199, SER200, HIS201, HIS202, TYR204, ASP205, ASP239, HIS244, ASP247, PHE286, ALA287, VAL288, GLN289, VAL290, PRO291, ALA292, MET293, ARG323, ASN325, VAL326, ASN328, GLU329, GLU354, TRP356, TYR374, GLU378, ILE381, SER419, HIS420, ASP421, THR422, ASP465, PRO466, and ARG469. PDB ID 3PXY in complex with Lapatinib has shown a few fluctuating residues; the most fluctuating residue is THR38-ARG53 and, in this case, some of the residues fluctuated beyond 15 Å. At the same time, many residues have formed interactions to make the complex stable, including SER74, SER75, ARG77, ARG108, GLU277, GLY279, GLY280, ALA281, MET329, ALA330, SER332, GLU369, VAL370, ARG388, GLY492, GLY493, LEU495, ALA496, ASN499, THR500, GLN548, ASN551, SER552, LEU553, ASN554, PHE555, ASP556, LEU557, LYS559, THR560, LYS585, ASP597, and LEU598. PDB ID 1E7U in complex with Lapatinib has shown none of the residues fluctuating beyond 2 Å. At the same time, many residues have formed interactions to make the complex stable, including ARG8, ASP25, GLY27, ALA28, ASP29, ASP30, GLY48, ILE50, PRO81, VAL82, ILE84, ARG8, LEU23, ASP25, GLY27, ALA28, ASP29, ASP30, VAL32, LYS45, MET46, ILE47, GLY48, ILE50, GLN58, LEU76, PRO81, VAL82, and ILE84 (Figure 6). The analysis of RMSF provides valuable insights into the stability and flexibility of protein-ligand complexes involving Lucidin and Lapatinib. In the context of various protein targets, Lucidin consistently demonstrates superior stability compared to Lapatinib, evidenced by lower fluctuations in critical residues. For the Estrogen Receptor (PDB ID 1A52), the RMSF analysis indicates minimal fluctuation in residues, with key residues like LEU327, MET343, and ARG394 forming stable interactions, suggesting the Lucidin complex maintains structural integrity, reinforcing its potential as a robust therapeutic agent. In contrast, the Lapatinib complex exhibited significant fluctuations, particularly in residues such as SER464 and LEU540-LEU544. This instability hints at a less favourable binding environment, possibly impacting therapeutic efficacy. In the case of Human HER2 (PDB ID-1N8Z), Lucidin shows residues like GLN135 and HIS164 with stable interactions, while fluctuations are mainly limited to SER147-PRO151, indicating that Lucidin establishes a well-defined binding site, enhancing the likelihood of sustained interaction. Conversely, the Lapatinib complex displays fluctuations in residues like SER47-GLU50 and LYS277-SER279, suggesting a less stable binding environment, which may result in weaker interactions and potential displacement in physiological conditions. Lucidin demonstrates superior stability and favourable binding characteristics compared to Lapatinib across various protein targets. The consistently lower RMSF values and stable interactions with crucial residues indicate that Lucidin forms more robust complexes, enhancing its potential as an effective therapeutic agent. These findings underscore the importance of selecting ligands that promote stability, as they are likely to yield improved pharmacological outcomes in drug design and development.

#### 2.5.3. Simulation Interaction Diagram (SID)

**Figure 7:**
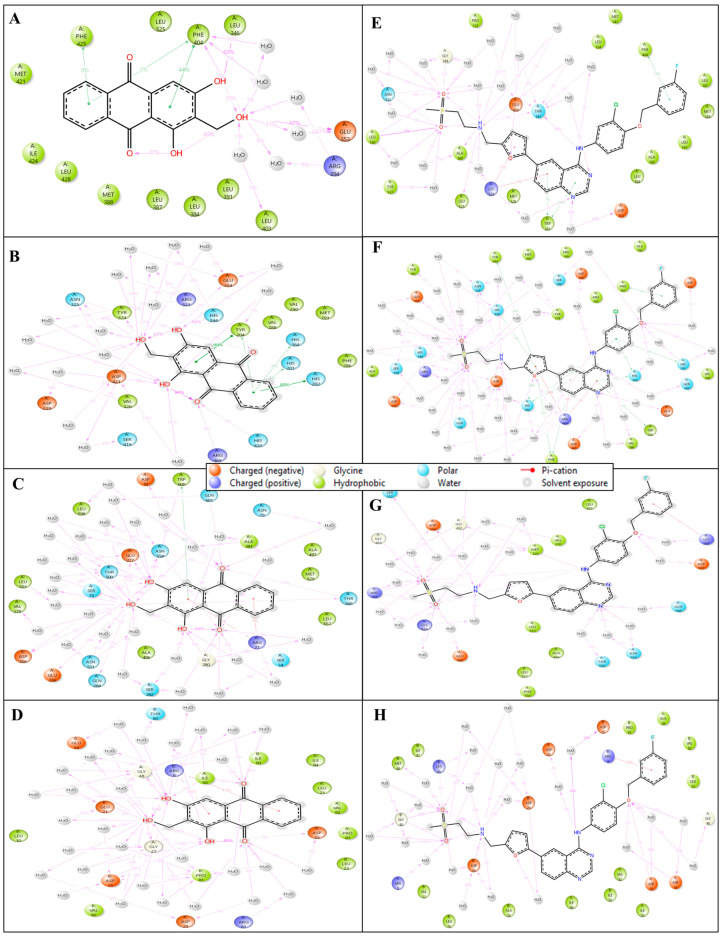
Showing the Simulation Interaction Diagram (SID) produced during 100 nanoseconds MD Simulation for PDB IDs (**A**) 1A52, (**B**) 1N8Z, (**C**) 3PXY, and (**D**) 1E7U in complex with Lucidin and (**E**) 1A52, (**F**) 1N8Z, (**G**) 3PXY, and (**H**) 1E7U in complex with control drug Lapatinib.

**Figure 8:**
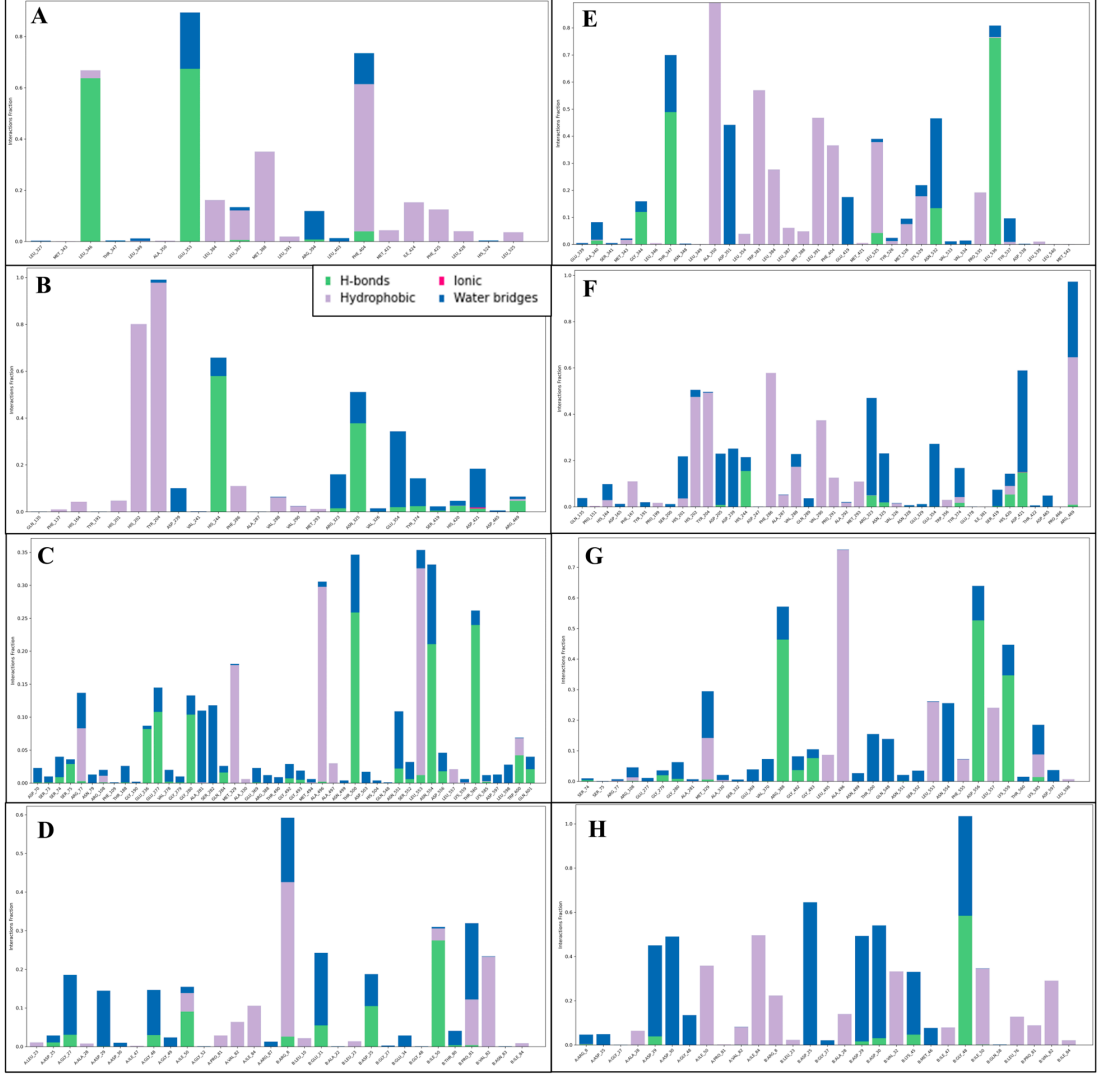
Showing the histogram representations for Simulation Interaction Diagram (SID) produced during 100 nanoseconds MD Simulation for PDB IDs (**A**) 1A52, (**B**) 1N8Z, (**C**) 3PXY, and (**D**) 1E7U in complex with Lucidin and (**E**) 1A52, (**F**) 1N8Z, (**G**) 3PXY, and (**H**) 1E7U in complex with control drug Lapatinib.

The SID visually represents protein-ligand interactions during molecular dynamics simulations. It includes labelled structures of proteins and ligands, highlighting binding sites and types of interactions. The interaction of Estrogen Receptor (PDB ID 1A52) with Lucidin formed hydrogen bonds among GLU353, LEU346, and PHE404 residues, and ARG394 residue with water molecules along two OH atoms; also, two π-π stacking contact PHE404 and PHE425 residues with two benzene rings of the ligand Lucidin also occur. The complex with protein Human HER2 (PDB ID 1N8Z) with Lucidin interacts with many hydrogen bonds among ASP239, GLU354, ARG323, ASP421, and TYR374 residues with water molecules, and HIS244 and ASN325 residues with three OH atoms. Four π-π stacking bonds interact along two benzene rings with HIS164, HIS201, HIS202, and TYR204 residues. In the interaction of Triphosphate Diphosphohydrolase 3 (NTPDase3) (PDB ID 3PXY) with Lucidin, many hydrogen bonds contact GLY280, THR560, ASN554, GLU277, TRP600, THR500, and GLU236 residues, and ALA281 and SER282 residues interact with water molecules along three OH atoms, while GLY280 and THR560 residues interact with two O atoms; moreover, a pi-cation bond interacts with the ARG77 residue and the benzene ring of the ligand. The complex of phosphoinositide 3-kinase (PDB ID 1E7U) and Lucidin interact with hydrogen bonds among ASP25, GLU21, ILE50 residues, and GLY27, ASP29, GLY48, THR80, PRO81 residues interact with water molecules along three OH atoms; also, two O atoms interact with ASP25, GLY27, and ASP29 residues with water molecules and ILE50 residue. Additionally, the ARG8 residue contacts two pi-cation bonds with two different benzene rings of the ligand. The interaction of Estrogen Receptor (PDB ID 1A52) with Lapatinib formed hydrogen bonds among the THR347 residue and ASN532 residue with water molecules along two NH atoms; the ASP351 residue along the N atom; GLY344, ASN532, LEU536 residue, and GLU419, TYR537, ALA340 residues with water molecules along two O atoms. Additionally, two π-π stacking contact TRP383 and PHE404 residues with two benzene rings and two pi-cation bonds interact with the LYS529 residue for two different benzene rings of the ligand Lapatinib. The complex with protein Human HER2 (PDB ID 1N8Z) with Lapatinib interact with several hydrogen bonds among ARG468 and ASP205 residues with water molecules, and ASP421 residue along two NH atoms; HIS164, HIS201, GLU354, ARG323, ASN325, ASP239, ASP421, and ARG469 residues interact with water molecules, and HIS244 residue interacts with four O atoms. Moreover, there are six π-π stacking bonds that contact PHE167, HIS202, TYR204, and PHE286 residue along four benzene rings; also, two pi-cations contact the ARG469 residue with two different benzene rings. In the interaction of CDK2 (PDB ID 3PXY) with Lapatinib, many hydrogen bonds contact LYS559 and MET329 residues with water molecules and ASP556 residue along the NH atom; the N atom interacts THR500, GLN548, and ASN554 residues with water molecules; LYS585, ASP556, and VAL370 residues with water molecules, and GLY493, ARG388, and LYS559 residues with three O atoms. Also, a pi-cation bond contacts the LYS585 residue along the benzene ring of the ligand. The complex of Phosphoinositide 3-kinase (PDB ID 1E7U) and Lapatinib interact with hydrogen bonds among ASP30, ASP29, LYS45, and ASP25 residues with water molecules along two NH atoms; also, three O atoms contact ASP29, ASP30, GLY48, MET46, and LYS45 residues with water molecules. Further, a pi-cation bond interacts with the ARG8 residue along the benzene ring of the ligand (Figure 7). Figure 8 shows the histogram for the count of interactions formed during the MD Simulation. Lucidin demonstrates stronger interactions than Lapatinib in several protein-ligand complexes, making it a better drug candidate. In the Estrogen Receptor complex (PDB ID 1A52), Lucidin forms multiple hydrogen bonds and π-π stacking interactions with key residues such as GLU353, LEU346, and PHE404, compared to Lapatinib’s fewer hydrogen bonds and interactions. Additionally, Lucidin forms robust hydrogen bonding and π-π stacking with Human HER2 (PDB ID 1N8Z) and NTPDase3 (PDB ID 3PXY), involving important residues like ASP239, HIS244, and THR560. This demonstrates better stability and binding efficiency than Lapatinib. Moreover, in the phosphoinositide 3-kinase (PDB ID 1E7U) complex, Lucidin engages in more hydrogen and pi-cation bonds, highlighting its enhanced affinity. These stronger interactions suggest Lucidin’s superior binding capability and potential as a more effective therapeutic agent.

## 3. Methods

We have performed several computational studies in this study, and a graphic abstract is shown to help us understand the studies better. The detailed methods are as follows.

### 3.1. Protein and Ligand Data Collection and Preparation

Data collection was initiated by searching in the RCSB database (https://rcsb.org/) (Accessed on 5 October 2024) and identifying the crucial proteins from breast cancer that resulted from ERα (PDB ID: 1A52), HER2 (PDB ID: 1N8Z), CDK2 (PDB ID: 3PXY), and PI3Kα (PDB ID: 1E7U) [15,16,17,18,24]. We imported this breast cancer protein to Schrodinger Maestro’s (https://schrodinger.com/, accessed on 5 October 2024) workspace and prepared it with Protein Preparation Workflow (PPW) [25,26]. The original structures of PDBID 1A52, 1N8Z, and 1E7U contain two chains of protein, native ligands, solvents, and others, while 3PXY contains four chains, ligands, and others [15,16,17,18]. In the PPW, we selected all four structures and capped termini, filled in the missing side chains, assigned bond orders, replaced hydrogens, created zero bond orders to metals and disulphide bonds, filled in the missing loops using Prime, and generated het stats with Epik at pH 7.4 ± 2 [25,27,28]. Further, in the optimisation tab, we sampled water orientations, used crystal symmetry, and optimised with PROPKA, and in the minimisation tab, we deleted water distant 5Å from the ligand and minimised all atoms using the Optimised Potentials for Liquid Simulations (OPLS4) forcefield [25,29,30]. After preparing the proteins, we kept them on Chain A with native ligands and deleted everything that may have created confusion during the calculation [15,16,17,18]. For the ligands, we downloaded the Natural Compounds library from the ZINC database (https://zinc.docking.org/, accessed on 5 October 2024), which is a curated collection of natural product-like compounds designed to aid in virtual screening and drug discovery. Hosted within the ZINC database, it offers various molecules that mimic naturally occurring compounds’ structural complexity and bioactivity. These compounds are derived from plants, microbes, and marine organisms, which have historically served as rich sources of therapeutic agents. The library is optimised for drug-like properties, including solubility, bioavailability, and synthetic feasibility, making it ideal for early-stage drug discovery. The ZINC Natural Products Library provides a valuable resource for identifying novel drug leads with high pharmacological potential by focusing on natural product-inspired molecules, and prepared it with the LigPrep tool in Maestro [9,25,31,32,33,34,35,36], where we browsed the file and filtered the compounds beyond 500 atoms and used the OPLS4 forcefield [25,29]. For the ionisation, we kept generating possible states at a target pH of 7 ± 2, used the Epik classing and desalt, and generated the tautomers [25,28]. For the stereoisomer computations, we retained specified chiralities, generating at most 32 per ligand and writing the outputs in an SDF file for further use [25].

### 3.2. Receptor Grid Generation and Multitargeted Docking and MM/GBSA Computation Strategies

Docking studies are crucial to identify drug candidates with minimal cost. To perform the docking studies, defining the grid area where the computations will be performed is essential. Grid generation in multitargeted docking involves creating a three-dimensional grid around the binding sites of multiple target proteins, such as ERα, HER2, CDK2, and PI3K, to identify potential binding regions for small molecules. This grid helps map the active sites and predict how ligands interact with different targets. A single ligand is simultaneously computationally screened against multiple protein targets in multitargeted docking. This approach aims to identify compounds that can bind effectively to all targets, increasing efficacy and reducing drug resistance. Multitargeted docking optimises drug design by addressing multiple cancer pathways simultaneously, enhancing therapeutic outcomes. We used Maestro’s Receptor Grid Generation tool to generate the grids [25,37]. In each of the cases, we had the native ligands, so we checked with the pick to identify the ligand molecule option and scaling factor of 1 with a partial charge cutoff of 0.25. In the site tab, we selected the centroid of the workspace ligands (which we selected from the workspace) and displayed the box area to understand the coverage and adjusted the dock ligands with length of 20 Å. Further, for the docking studies, we used the Virtual Screening Workflow (VSW) tool, wherein, in the input tab, we browsed the source of ligands (prepared library) and generated unique properties to remove any duplicate ligands [25,38]. In the Filtering tab, we kept running the QikProp on each structure to generate the pharmacokinetics and passed it to Lipinski’s rule to filter them better [25,39,40,41,42]. The preparation tab was left as we already had the prepared structures, and in the receptor tab, we browsed the grid file and moved it to the docking tab. In the docking tab, we checked to enhance the planarity of conjugated pi groups, used Epik state penalties for docking and a scaling factor of 0.80, and kept the partial charge to 0.15 [25,28]. For the docking, we checked High Throughput Virtual Screening (HTVS), Standard Precision (SP), and Extra Precise (XP) docking. Only the top 10% of HTVS outputs were passed to SP, and the top 10% of SP were passed to XP. In the XP, we kept generating four poses per compound and passed 100% to Molecular Mechanics Generalized Born Surface Area (MM/GBSA) for binding free energy-based pose filtering. Further, we downloaded and prepared one FDA-approved compound, Lapatinib, to compare our results, and we performed the docking with the same parameters. After the docking studies, we exported all the results to CSV and analysed them to identify multitargeted compounds with better efficacy than the Lapatinib compound.

### 3.3. Molecular Interaction Fingerprints and Pharmacokinetics

Molecular Interaction Fingerprints (MIFs) are a computational tool used to represent the interactions between a ligand (such as a drug candidate) and a target protein at the molecular level. They convert complex interaction data into a simplified binary or numerical format, where each bit or number indicates the presence or absence of specific types of interactions, such as hydrogen bonds, hydrophobic contacts, van der Waals forces, or electrostatic interactions [12,20]. MIFs are valuable for comparing and analysing how different ligands interact with the same protein or how a single ligand interacts with different proteins. They also help identify key interaction patterns contributing to binding affinity and specificity. In multitargeted drug design, MIFs can be used to find ligands that form optimal interactions across multiple protein targets, enhancing drug efficacy and reducing the likelihood of resistance by ensuring the ligand can engage with multiple key proteins involved in disease pathways. This approach aids in refining drug candidates for better-multitargeted activity. We used the Interaction Fingerprints tool in Maestro, where we checked the receptor-ligand complexes option and went further with any contact option and aligned sequences as we had multiple protein complexes. We generated the fingerprints and displayed them in the form of a matrix. N to C terminals were highlighted with different colours [12,20,25]. The count of residue and ligand interactions was also plotted to clarify and remove non-interacting residues. Further, the pharmacokinetic properties, including ADMET (Absorption, Distribution, Metabolism, and Excretion, and Toxicity) parameters, were predicted using Schrödinger’s QikProp tool and Lipinski’s rule [25,39,40,41,42]. Pharmacokinetics, often encapsulated by the acronym ADMET (Absorption, Distribution, Metabolism, Excretion, and Toxicity), refers to the comprehensive study of how a drug behaves within the body. Understanding these factors is essential for developing safe and effective drugs. Absorption describes how the drug enters the bloodstream after administration, influenced by solubility and the ability to cross biological membranes. Once in the bloodstream, distribution explains how the drug spreads to various tissues and organs, depending on blood flow, tissue affinity, and protein binding. Metabolism primarily occurs in the liver, where enzymes modify the drug into active or inactive metabolites, particularly those in the cytochrome P450 family. These metabolites and the parent drug are then excreted from the body, usually through urine (kidneys) or faeces (bile). Finally, toxicity is a critical factor that evaluates the drug’s potential harmful effects, including organ damage, off-target effects, and adverse reactions. Optimising ADMET properties is crucial to ensuring a drug is safe and effective. In multitargeted drug design, favourable ADMET profiles help ensure that drugs remain active in the body long enough to exert their effects on multiple targets while minimising side effects and resistance. These generated properties of the identified multitargeted compound, Lapatinib, and the standard values were compared to provide a clear understanding [12,20,25].

### 3.4. WaterMap Analysis

WaterMap is a computational tool used in drug discovery to analyse the role of water molecules in binding ligands to target proteins. It helps researchers understand how water molecules in and around a protein’s binding site influence ligand binding, which is critical for optimising drug design. It identifies and characterises water molecules within the binding site based on their energetic properties. Water molecules that are tightly bound and energetically favourable might support the protein-ligand interaction, while others, known as “unfavourable” water molecules, can be displaced by a well-designed drug molecule to enhance binding affinity [12]. WaterMap provides insights into how modifying a ligand to interact with or displace specific water molecules can strengthen its binding to the target protein after mapping the water molecules and improve drug efficacy by enhancing ligand binding and optimising drug-target interactions. In multitargeted drug design, WaterMap can be especially useful in fine-tuning the binding of a single ligand to multiple proteins by optimising how it engages with water molecules in different binding sites and leads to more potent drugs with higher specificity and reduced chances of resistance. We have used the WaterMap-perform computation tool in Maestro to perform the computations, which uses the GPU in the backend for computations (MD Simulation) [25,43]. The binding site definition was kept to the ligand by selecting it from the workspace and analysing the water molecules within the 10 Å of selected atoms (ligand). For the simulation setup, we kept the truncating protein using the OPLS4 force field and treated the existing (if any) water molecules as solvents [25,29]. The simulation time was kept to 5 nanoseconds in each case and kept returning the trajectories. Further, for the analysing the results, we used the WaterMap-examine Results panel, where we selected the complex from the workspace and kept displaying the receptor, ligand, H-bonds, and markers, and analysed the bonds and various energies, including the enthalpy, entropy, and free energy, and plotted the tables and figures.

### 3.5. Molecular Dynamics Simulations Studies

Molecular Dynamics (MD) Simulation is a powerful computational technique used to study the physical movements of atoms and molecules over time, providing insights into the dynamic behaviour of biological systems, such as proteins, nucleic acids, and drug interactions. In drug discovery, MD simulations allow researchers to model how a drug (ligand) interacts with its target protein in a dynamic, real-world environment rather than a static structure. It works by solving Newton’s equations of motion for a system of atoms, allowing scientists to observe how the structure of a protein changes and how a ligand moves within its binding site. This method helps understand the protein-ligand complex’s flexibility, revealing important interactions that may be missed in static docking studies. MD also allows for studying key factors like conformational changes, binding stability, and solvent (such as water) and temperature effects on the binding process. We used the Desmond Package in Schrodinger Maestro, developed by DE Shaw Research [25,43], to perform the MD Simulation studies in three steps: preparing the system files, running the production, and analysing the results. The System Builder panel in Maestro was used to prepare the system files where we used the Transferable Intermolecular Potential with 3 Points (TIP3P) water model; the boundary conditions were kept in Orthorhombic shape in a buffer with distances of 10 × 10 × 10 Å from the protein surface and we minimised the volume of the shape to fit on the protein-ligand complex surface [25,44]. The ions and salt placement within 20Å were excluded, and we added 7Na^+^, 9Na^+^, 4Na^+^, and 7Cl^−^ ions in the protein-ligand complex of PD BIDs 1A52, 1N8Z, 3PXY, and 1E7U to neutralise the system, followed by using the OPLS4 force field [15,16,17,18,25,29,45,46]. For the Production Run, we used the Molecular Dynamics panel, where we loaded the prepared system builder file from the workspace and kept the simulation time to 100 nanoseconds, elapsed to 0, recording intervals for trajectories to every 100 picoseconds with an energy level of 1.2 to generate a total of 1000 frames in each case. For the ensemble class, we used the isothermal–isobaric ensemble (constant temperature and constant pressure ensemble) and the NPT ensemble and relaxed the system before production [25,47]. The production produced the recorded trajectories, which were then analysed with Maestro’s Simulation Interaction Diagram (SID) tool to understand deviation, fluctuations, and intermolecular interactions in each case [25].

## 4. Conclusions

Breast cancer remains a significant global health challenge, accounting for a considerable number of cancer diagnoses and deaths among women, and the complexity necessitates the development of multitargeted therapeutic strategies that enhance treatment efficacy while minimising adverse effects. Our multitargeted docking study has shown far improved results for the identified drug candidate Lucidin from the *Rubia cordifolia* plant, which showed almost 1.5–2-fold better results than the control drug Lapatinib, with docking scores of −5.66 to −9.91 for Lucidin and −3.57 to −7.97 for Lapatinib. Also, pharmacokinetics surpasses the criteria to fit into a better breast cancer drug, along with the diverse interaction types, including leucine, methionine, isoleucine, valine, alanine, proline, phenylalanine, and tryptophan, revealed using MIFs. The 5 nanoseconds WaterMap study has also confirmed that Lucidin has a better potency than the control drug, and the same was also validated by the 100 nanoseconds MD Simulation, which showed that the deviation and fluctuations were mostly under control, and many intermolecular interactions supported the fact that the complex had a stable performance. Our study supports the fact that the complexes are stable and that Lucidin is an improved multitarget drug candidate for breast cancer.

## Figures and Tables

**Table 1:** Showing the Energies Produced (Kcal/mol) During Protein Preparation for each PDB IDs.

Energy Parameters	1A52	3PXY	1E7U	1N8Z
Total energy of the system	−1.00 × 10^3^	−2.59 × 10^3^	−7.87 × 10^2^	−2.79 × 10^3^
Total Potential Energy	−1.00 × 10^3^	−2.59 × 10^3^	−7.87 × 10^2^	−2.79 × 10^3^
Total Kinetic Energy	0.00	0.00	0.00	0.00
Temperature of the system (K)	0	0	0	0
Bond Stretch Energy	1.13 × 10^2^	2.64 × 10^2^	8.68 × 10^1^	2.75 × 10^2^
Angle Bending Energy	5.05 × 10^2^	1.21 × 10^3^	4.00 × 10^2^	1.28 × 10^3^
Torsion Angle Energy	3.52 × 10^2^	9.83 × 10^2^	4.16 × 10^2^	1.23 × 10^3^
Restraining Energy for Torsions	0.00	0.00	0.00	0.00
1,4 Lennard Jones Energy	1.14 × 10^3^	2.68 × 10^3^	8.63 × 10^2^	2.84 × 10^3^
1,4 Electrostatic Energy	4.38 × 10^2^	8.33 × 10^2^	3.78 × 10^2^	7.85 × 10^2^
Lennard Jones Energy	−2.30 × 10^3^	−5.59 × 10^3^	−1.89 × 10^3^	−6.15 × 10^3^
Electrostatic Energy	−1.29 × 10^3^	−3.06 × 10^3^	−1.05 × 10^3^	−3.11 × 10^3^
H-bond Energy	0.00	0.00	0.00	0.00

**Table 2:** Showing the considered PDBIDs and docked ligand names (our identified multitargeted candidate—Lucidin), FDA-approved (Lapatinib), and other energies produced during the docking studies.

**PDBIDs**	**Molecule**	**Resolution**	**Bend Energy** **-S-OPLS**	**Potential Energy** **-S-OPLS**	**RMS Derivative** **-S-OPLS**	**Gridbox Xcent**	**Gridbox** **Ycent**	**Gridbox Zcent**
1A52	Lucidin	2.80	504.95	−836.69	0.09	106.69	14.70	96.26
1N8Z	Lucidin	2.20	1277.78	−2596.37	0.09	36.06	55.44	60.72
3PXY	Lucidin	2.85	1207.80	−2251.63	0.11	41.18	13.25	−38.90
1E7U	Lucidin	1.89	399.41	−745.47	0.09	70.79	56.83	16.75
1A52	Lapatinib	2.80	504.95	−836.69	0.09	106.69	14.70	96.26
1N8Z	Lapatinib	2.20	1277.78	−2596.37	0.09	36.06	55.44	60.72
3PXY	Lapatinib	2.85	1207.80	−2251.63	0.11	41.18	13.25	−38.90
1E7U	Lapatinib	1.89	399.41	−745.47	0.09	70.79	56.83	16.75
**PDBIDs**	**Molecule**	**XP GScore**	**XP HBond**	**Docking Score**	**Glide Ligand** **Efficiency ln**	**Glide Ligand** **Efficiency sa**	**Complex** **vdW**	**MMGBSA dG Bind**
1A52	Lucidin	−9.98	−1.55	−9.91	−2.48	−1.35	−1066.30	−46.51
1N8Z	Lucidin	−6.67	−0.67	−6.60	−1.65	−0.90	−3236.45	−25.12
3PXY	Lucidin	−5.73	−1.81	−5.66	−1.42	−0.77	−2752.87	−31.82
1E7U	Lucidin	−7.70	−3.02	−7.62	−1.91	−1.03	−958.29	−44.92
1A52	Lapatinib	−7.32	0.00	−7.32	−1.56	−0.63	−1065.28	−38.51
1N8Z	Lapatinib	−3.57	−0.78	−3.57	−0.76	−0.31	−3259.98	−28.39
3PXY	Lapatinib	−3.62	0.00	−3.62	−0.77	−0.31	−2775.54	−43.31
1E7U	Lapatinib	−7.97	−0.56	−7.97	−1.70	−0.68	−983.73	−58.84

**Table 3:** Showing the pharmacokinetics computed with the QikProp tool in Maestro.

Title	Standard Values	Lucidin	Lapatinib
#acid	0–1	0	0
#amide	0–1	0	0
#amidine	0	0	0
#amine	0–1	0	1
#in34	N/A	0	0
#in56	N/A	14	27
#metab	1–8	3	6
#NandO	2–15	5	8
#noncon	N/A	0	0
#nonHatm	N/A	20	40
#ringatoms	N/A	14	27
#rotor	0–15	4	10
#rtvFG	0–2	0	0
#stars	0–5	0	2
% HumanOralAbs	>80% is high, <25% is poor	65.73	80.087
accptHB	2.0–20.0	6.2	8.25
ACxDN^.5/SA	0.0–0.05	0.0185614	0.0094773
CIQPlogS	−6.5–0.5	−3.159	−8.299
CNS	−2 (inactive), +2 (active)	−2	1
dip^2/V	0.0–0.13	0.0091892	0.0521805
dipole	1.0–12.5	2.712	9.289
donorHB	0.0–6.0	2	1
EA(eV)	−0.9–1.7	1.369	1.236
FISA	7.0–330.0	212.17	96.007
FOSA	0.0–750.0	43.581	200.733
glob	0.75–0.95	0.8823879	0.7769133
HumanOralAbs	N/A	3	1
IP(eV)	7.9–10.5	9.426	8.19
Jm	N/A	0.067	0
mol MW	130.0–725.0	270.241	581.06
PISA	0.0–450.0	216.632	480.366
PSA	7.0–200.0	115.139	98.987
QPlogBB	−3.0–1.2	−1.494	−0.472
QPlogHERG	concern below −5	−4.468	−8.159
QPlogKhsa	−1.5–1.5	−0.452	1.014
QPlogKp	−8.0–−1.0	−4.283	−2.699
QPlogPC16	4.0–18.0	9.179	18.017
QPlogPo/w	−2.0–6.5	0.56	5.914
QPlogPoct	8.0–35.0	14.903	26.617
QPlogPw	4.0–45.0	11.926	13.152
QPlogS	−6.5–0.5	−2.324	−6.499
QPPCaco	<25 poor, >500 great	96.358	303.643
QPPMDCK	<25 poor, >500 great	39.451	490.161
QPpolrz	13.0–70.0	25.369	58.195
RuleOfFive	maximum is 4	0	2
RuleOfThree	maximum is 3	0	1
SAamideO	0.0–35.0	0	0
SAfluorine	0.0–100.0	0	46.545
SASA	300.0–1000.0	472.384	870.5
Type	N/A	small	small
volume	500.0–2000.0	800.174	1653.729
WPSA	0.0–175.0	0	93.394

## Data Availability

The data is already provided in the manuscript’s table and figures section.
